# The unremarkable alveolar epithelial glycocalyx: a thorium dioxide-based electron microscopic comparison after heparinase or pneumolysin treatment

**DOI:** 10.1007/s00418-023-02211-7

**Published:** 2023-06-29

**Authors:** Sara Timm, Marie Lettau, Jan Hegermann, Maria Linda Rocha, Sarah Weidenfeld, Diana Fatykhova, Birgitt Gutbier, Geraldine Nouailles, Elena Lopez-Rodriguez, Andreas Hocke, Stefan Hippenstiel, Martin Witzenrath, Wolfgang M. Kuebler, Matthias Ochs

**Affiliations:** 1 Core Facility Electron Microscopy, Charité-Universitätsmedizin Berlin, 13353 Berlin, Germany; 2 Institute of Functional Anatomy, Charité-Universitätsmedizin Berlin, 10115 Berlin, Germany; 3 Research Core Unit Electron Microscopy and Institute of Functional and Applied Anatomy, Hannover Medical School, 30625 Hannover, Germany; 4 Institute of Pathology, Vivantes Klinikum im Friedrichshain, 10249 Berlin, Germany; 5 Institute of Physiology, Charité-Universitätsmedizin Berlin, 10117 Berlin, Germany; 6 Department of Infectious Diseases, Respiratory Medicine and Critical Care, Charité-Universitätsmedizin Berlin, 10117 Berlin, Germany; 7 German Center for Lung Research (DZL), Berlin, Germany

**Keywords:** Alveolar epithelial glycocalyx, Thorium dioxide, Heparinase, Pneumolysin, Electron tomography, Lung stereology

## Abstract

Recent investigations analyzed in depth the biochemical and biophysical properties of the endothelial glycocalyx. In comparison, this complex cell-covering structure is largely understudied in alveolar epithelial cells. To better characterize the alveolar glycocalyx ultrastructure, unaffected versus injured human lung tissue explants and mouse lungs were analyzed by transmission electron microscopy. Lung tissue was treated with either heparinase (HEP), known to shed glycocalyx components, or pneumolysin (PLY), the exotoxin of *Streptococcus pneumoniae* not investigated for structural glycocalyx effects so far. Cationic colloidal thorium dioxide (cThO_2_) particles were used for glycocalyx glycosaminoglycan visualization. The level of cThO_2_ particles orthogonal to apical cell membranes (≙ stained glycosaminoglycan height) of alveolar epithelial type I (AEI) and type II (AEII) cells was stereologically measured. In addition, cThO_2_ particle density was studied by dual-axis electron tomography (≙ stained glycosaminoglycan density in three dimensions). For untreated samples, the average cThO_2_ particle level was ≈ 18 nm for human AEI, ≈ 17 nm for mouse AEI, ≈ 44 nm for human AEII and ≈ 35 nm for mouse AEII. Both treatments, HEP and PLY, resulted in a significant reduction of cThO_2_ particle levels on human and mouse AEI and AEII. Moreover, a HEP- and PLY-associated reduction in cThO_2_ particle density was observed. The present study provides quantitative data on the differential glycocalyx distribution on AEI and AEII based on cThO_2_ and demonstrates alveolar glycocalyx shedding in response to HEP or PLY resulting in a structural reduction in both glycosaminoglycan height and density. Future studies should elucidate the underlying alveolar epithelial cell type-specific distribution of glycocalyx subcomponents for better functional understanding.

## Introduction

First mentions of an "externous coat" around cells go back almost a century, followed by decades of histochemical methods to reveal its morphology (Martins and Bairos 2002). In the early 1960s, Bennett proposed the term “glycocalyx “ (derived from Greek: glykys = sweet, kalyx = husk) (Bennett 1963), which perfectly describes a main structural feature: a carbohydrate-enriched layer coating nearly all free parts of cell membranes. More recent research classifies glycocalyx glycans depending on their linkage to proteins or lipids into proteoglycans, glycoproteins, glycolipids, and free glycans (Mockl 2020). For many cell types, including both alveolar epithelial type I (AEI) and type II (AEII) cells, mass ratios of the different glycocalyx subcomponents are not available so far. However, there has been growing recognition that glycosaminoglycans (GAGs) constitute a major component being responsible for many common glycocalyx stainings due to their negative charge properties. Glycocalyx GAGs are long, linear glycans that can be found covalently bound to core proteins, constituting the class of proteoglycans, whereas heparan and chondroitin sulfate represent classical bound GAGs on the alveolar epithelium (Haeger et al. 2018; Bray 2001). The more simple non-protein non-sulfated glycan hyaluronan makes up a further major alveolar glycocalyx GAG (Bray 2001).

In general, GAGs present on cell surfaces and in the extracellular matrix are known to regulate a variety of biophysical and biochemical tissue properties, whereby alterations in their distribution or composition are often associated with major pathophysiological changes in cells and organs (Morla 2019; Gesslbauer et al. 2013; Wang and Chi 2022). The specific functions of lung epithelial glycocalyx GAGs, especially those on AEI and AEII, are relatively unexplored despite their important localization at the immediate interface between organism and environment. Growing evidence implicates alveolar GAGs in air–blood barrier permeability, surfactant and aqueous hypophase homeostasis, pathogen binding as well as alveolar development and injury (Ochs et al. 2020; Rizzo et al. 2022; Martins and Bairos 2002; Weidenfeld and Kuebler 2018; Haeger et al. 2016, 2018; LaRiviere et al. 2020; Wigen et al. 2019; Rizzo and Schmidt 2023). To date, however, there are still no sufficient quantitative and qualitative microscopic data on the alveolar epithelial glycocalyx GAG distribution in physiological and pathophysiological settings. Thus, there is a lack of reference values for future studies which should better reveal in depth the morphological and functional properties of the alveolar epithelial glycocalyx. Hence, the present study aimed to characterize the ultrastructurally unremarkable and affected alveolar glycocalyx by transmission electron microscopy (TEM). To this end, we treated human lung explants and mouse lungs with either heparinase (HEP) as an established glycocalyx-shedding enzyme or with pneumolysin (PLY), a bacterial exotoxin known for its ability to induce lung injury but unrelated to structural alveolar epithelial glycocalyx changes so far.

### Heparinase-specific shedding of alveolar epithelial glycocalyx

Given the close association of acute lung injury with alveolar glycocalyx degradation (Rizzo et al. 2022; Haeger et al. 2016, 2018; LaRiviere et al. 2020), selective GAG shedding provides a useful reference model for collecting ultrastructural data for comparison with PLY. Bacterial HEP enzymes specifically degrade heparan sulfate and heparin chains (Boyce and Walsh 2022). The GAG heparan sulfate is not only a common proteoglycan component on the alveolar epithelium, but is also present on almost all cell types as well as in basement membranes and extracellular matrices in various tissues due to its general involvement in physiological activities (Hayashida et al. 2022; Collins and Troeberg 2019). The synthesis and degradation of heparan sulfate represents a critical and sensitive, tightly controlled process. Of note, heparan sulfate can be depolymerized by the mammalian enzyme heparanase, which not only ensures continual provision of effective synthesis products under physiological conditions, but is also a major regulator in pathophysiological processes such as inflammation, sepsis, and cancer (Boyce and Walsh 2022; Collins and Troeberg 2019; Wu and Davies 2020; Sanderson et al. 2017).

### Pneumolysin—unknown effects on alveolar epithelial glycocalyx

*Streptococcus pneumoniae* invasion is a major cause of pneumonia, sepsis, and meningitis worldwide, being a leading cause of death especially among infants, young children, and patients with multi-morbidity (Briles et al. 2019). This bacterium produces the pore-forming virulence factor PLY, which favors its penetration into the interstitium and dissemination into the bloodstream (Witzenrath et al. 2006b; Rubins et al. 1995; Woodhead 2002; Cockeran et al. 2002). In addition, PLY might separate tight junctions (Rayner et al. 1995). More recent research has shown interactions with several other host molecules in a pro- and anti-inflammatory manner (Pereira et al. 2022). Alveolar epithelial glycocalyx alteration by PLY would be a conceivable further effect related to lung injury, but has not been investigated so far.

### Cationic hydrous thorium dioxide colloids—glycocalyx visualization

To date, research has shown that the alveolar lining layer mainly consists of water, surfactant, and glycocalyx components. In contrast to surfactant, visualizing the delicate, dynamically organized glycocalyx is a challenging task, whereby its detailed architecture can only be resolved at the TEM level (Ochs et al. 2020). Cryoprocessing might be most capable of preserving the alveolar epithelial glycocalyx in its native state. However, current protocols for fixing native lung tissue by high pressure freezing do not provide a suitable approach for quantitative stereological TEM analyses since most alveoli collapse. Therefore, the current study evaluated chemically fixed, conventionally embedded lung samples. Many staining reagents have been used over the decades of glycocalyx studies. Best known are certainly ruthenium red and alcian blue. In addition, colloidal iron, phosphotungstic acid, lanthanum nitrate, or lectins (concanavalin A, wheat germ agglutinin, and peanut agglutinin) were used as well (Ochs et al. 2020). Staining with cationic hydrous thorium dioxide colloids (cThO_2_) dates back to the late 1920s, where it was used as the X-ray contrast medium “Thorotrast” (Dickson 1932) until carcinogenic effects became known (Stover 1983). Half a century later, cThO_2_ was rediscovered and made available as a useful contrast agent for TEM due to its capacity to bind acid GAGs (Groot 1981). In comparison to other staining reagents like alcian blue, ruthenium red, and positive colloidal iron, cThO_2_ particles with a size of around 1 to 1.7 nm show better penetration into the tissue and higher electron density easily detected by TEM (Groot 1981; Hegermann et al. 2016; Lünsdorf et al. 2006). But despite these advantages, cThO_2_ remains less suitable for routine examinations, which is attributed to its radioactivity that requires strict approval as well as careful handling. CThO_2_ treatment of fixed samples prior to embedding also allows for three-dimensional (3D) analysis using electron tomography (ET).

## Material and methods

### Human lung tissue, treatment, and fixation

Fresh human lung tissue explants were obtained from two female patients (52 and 78 years old) suffering from lung carcinoma, who underwent lung resection at local thoracic surgeries. Both patients had a history of chronic obstructive pulmonary disease (patient 1 in early and patient 2 in advanced stage). The study was approved by the ethics committee (Charité-Universitätsmedizin Berlin, Germany, EA2/079/13). Written informed consent was obtained from both patients.

Macroscopically unremarkable (tumor-free) peripheral human lung tissue was dissected into small pieces (1 mm × 1 mm × 3 mm) and incubated for 1 h at 37 °C in Roswell Park Memorial Institute (RPMI) 1640 medium (Gibco by Life Technologies, Carlsbad, CA, USA) either with HEP (2 U/400 µl) (heparinase I and III blend from *Flavobacterium heparinum*, H3917, Sigma-Aldrich, St. Louis, MO, USA) (*n* = 2 from patient 1, *n* = 1 from patient 2) or with PLY (0.08 µg/400 µl) (provided by Timothy Mitchell; Mitchell et al. 1989) (*n* = 2 from patient 1, *n* = 1 from patient 2). For comparison with untreated human lung, tissue pieces (*n* = 2 from patient 1, *n* = 1 from patient 2) were incubated for 1 h in pure RPMI medium (400 µl). Subsequently, all tissues were fixed and stored in 0.15 M HEPES (Roth, Karlsruhe, Germany) (pH 7.35) containing 1.5% paraformaldehyde and 1.5% glutaraldehyde (both from Serva, Heidelberg, Germany).

### Mouse lung tissue, treatment, and fixation

Nine female C57Bl/6 mice were housed under specific-pathogen-free conditions with free dietary access for 16 to 19 weeks before treatment. Animal procedures were approved by institutional authorities (“Tierschutzbeauftragte” and “Tierschutzausschuss” of the Charité—Universitätsmedizin Berlin, Germany) and local governmental authorities (State Office for Health and Social Affairs Berlin, Germany).

Mice were anesthetized and heparinized with Ketamin (165–250 mg/kg body weight) and Xylazin (15–25 mg/kg body weight) diluted in 0.9% sodium chloride (NaCl) solution with 50% heparin (5000 IU/ml). After laparotomy and exsanguination by cutting the vena cava caudalis, animals were tracheotomized and ventilated. Following sternotomy and cannulation of the left atrium and pulmonary artery, the lungs were perfused with 37 °C sterile Krebs–Henseleit hydroxyethylamylopectin buffer (1 ml/min) (Serag-Wiesner AG, Naila, Germany) and ventilated by negative pressure (Pexp −4.5, Pins −9.0 cmH_2_O) in a humidified chamber (Witzenrath et al. 2006a). To ensure stable experimental conditions, a 20-min baseline was performed before stimulation (e.g., to control chamber pressure, tidal volume, and pulmonary artery pressure). Then, an intratracheal bolus of either HEP in 0.9% NaCl solution (0.625 U/25 µl) (*n* = 3) or PLY in 0.9% NaCl solution (1 µg/25 µl) (*n* = 3) or distilled water in 0.9% NaCl solution (0.15 µl/25 µl) (*n* = 3) was aerosolized with a microsprayer (Penn-Century, Wyndmoor, PA, USA). Mice were ventilated for further 30 min. This time period was chosen because PLY stimulation is known to produce marked hyperpermeability of the pulmonary barrier after 30 min of intratracheal aerosolization (Witzenrath et al. 2006b). Then, mouse lungs were removed and immediately fixed by intratracheal instillation with 0.15 M HEPES (pH 7.35) containing 1.5% paraformaldehyde and 1.5% glutaraldehyde under pressure of a 20 cmH_2_O. Lungs were removed, stored in the fixative at 4 °C for 1 week, and cut into small pieces (1 mm × 1 mm × 1 mm). Three pieces each were randomly selected from all mouse lung samples.

### Staining with ThO_2_ particles, embedding, and sectioning

To increase the specificity of negatively charged glycocalyx components, lung tissue pieces were immersed in a low-pH solution (pH 3) of 100 mM sodium acetate buffer for 5 min, followed by incubation in 0.5% cThO_2_ in sodium acetate for 5 min and again in sodium acetate for 5 min. During every step, samples were gently massaged with a wooden skewer to allow the solutions to be distributed in the alveoli. After the cThO_2_ treatment, samples were stored in 0.15 M HEPES (pH 7.35) containing 1.5% paraformaldehyde and 1.5% glutaraldehyde until further processing. Samples were osmicated with 1% OsO_4_ (Electron Microscopy Sciences, Hatfield, PA, USA) in 0.1 M cacodylate buffer for 2 h at room temperature followed by incubation in half-saturated (4%) aqueous uranyl acetate (Merck, Burlington, MA, USA) over night at 4 °C. Between the solution-changing steps, samples were washed with 0.15 M HEPES and 0.1 M cacodylate buffer. After dehydration in a graded acetone series, the samples were transferred to Epon resin (Serva, Heidelberg, Germany).

For quantitative stereological two-dimensional TEM analysis, ultra-thin sections (70 nm) of all samples were prepared with an Ultra 45° diamond knife (DiATOME, Nidau, Switzerland) at an ultramicrotome (Leica UltraCut S, Wetzlar, Germany) and contrasted with Reynolds' lead citrate (Reynolds 1963) (Merck, Burlington, MA, USA). For qualitative ET analysis, semi-thin sections (250 nm) of control and treated human tissue explants were prepared and cut with an Ultra Semi diamond knife (DiATOME, Nidau, Switzerland) as previously described in Lettau et al. ( 2022). Sections were contrasted with Reynolds’ lead citrate, and for tilt series alignment, incubated with fiducial 10 nm standard gold nanoparticles (Cytodiagnostics, Burlington, ON, Canada) from both sides for 3 min each and finally dried with filter paper.

###  Stereological measurements and statistical analysis

**Fig. 1:**
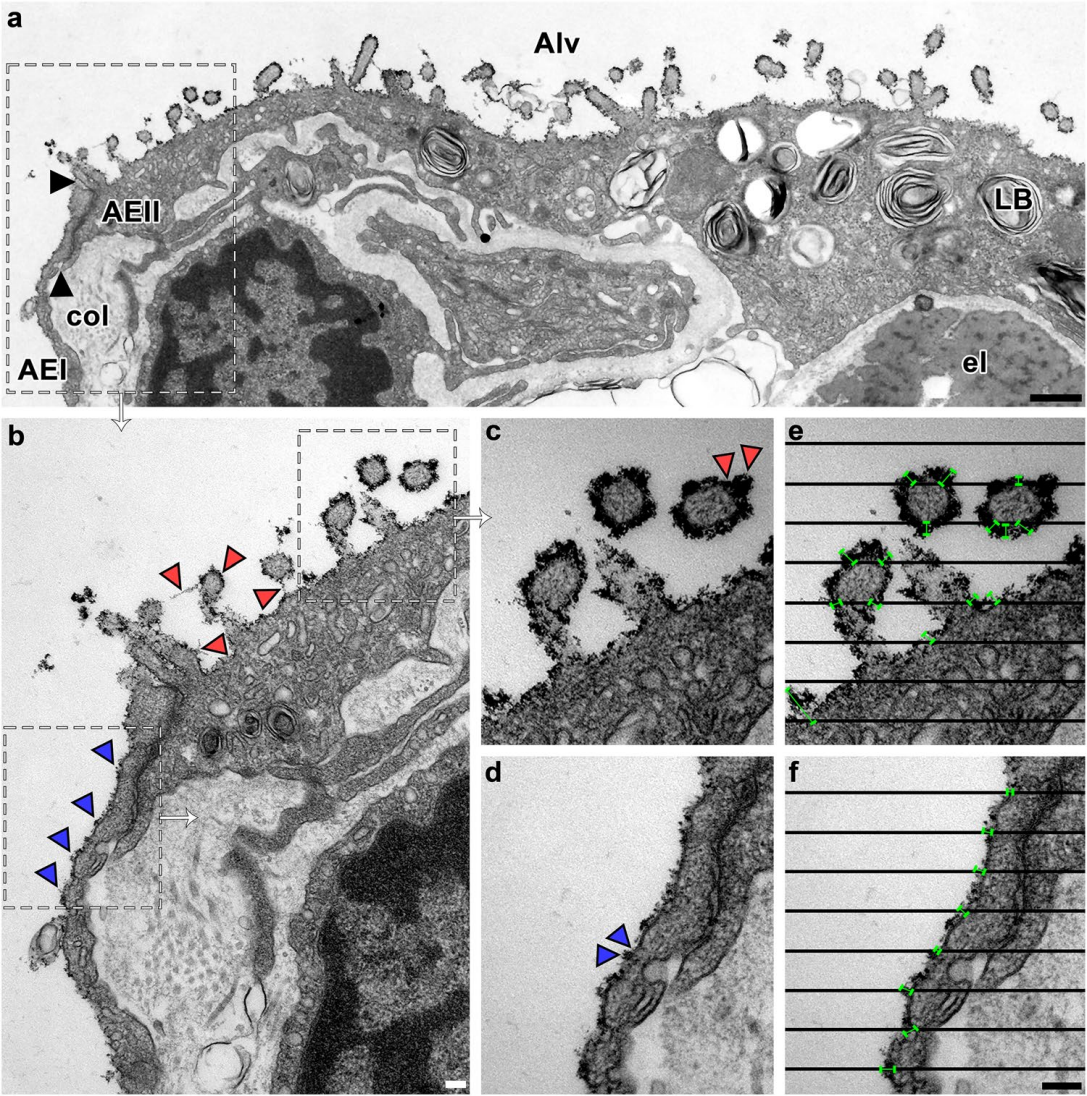
Measurement of alveolar epithelial cThO_2_ particle levels. TEM of an ultra-thin section from human control lung tissue. **a** AEI and AEII facing the alveolar lumen (Alv) with thin, dark cThO_2_ particle layer on apical membranes. Black arrows mark the boundary between AEI and AEII, shown in **b** at higher magnification. LB = lamellar body, col = collagen fibers, el = elastic fibers. Scale bar: 500 nm. **b** Magnified boundary between AEI and AEII from **a**. Blue arrowheads indicate a continuous cThO_2_ layer on AEI. Red arrowheads indicate a continuous cThO_2_ layer on AEII, some of which have filamentous extensions. Scale bar: 100 nm. **c**–**f** Magnified areas from **b**. Note the intense cThO_2_ staining on AEII in **c** with locally larger extensions (red arrowheads) versus the less intense, thinner cThO_2_ staining on AEI in d with less large extensions (blue arrowheads). The black line grid in **e** and **f** is placed over **c** and **d** images. Orthogonal distance from clearly visible cell membranes intersected by a line to the end of cThO_2_ staining was measured (green bars). Cut membranes that were not clearly identifiable due to orientation or overlap within the section were not included in the analysis. Scale bar: 100 nm

The investigator was blinded for work at the microscope and measurements. Examination of the grids were carried out with a Zeiss Leo 906 electron microscope at 80 kV acceleration voltage (Zeiss, Oberkochen, Germany) equipped with a slow-scan 2 K charge-coupled device (CCD) camera (TRS-Tröndle, Moorenweis, Germany) with associated software Image SP (version 1.2.890). The regions of interest were the apical membranes of human and mouse AEI and AEII. The field of view was defined to the rules of systematic uniform random area sampling (Tschanz et al. 2014). All images for measurements were prepared in the same ×35.970 on-microscope magnification. The measurements of cThO_2_ particle levels (≙ stained glycosaminoglycan height) were planned as orthogonal intercept lengths (Jensen et al. 1979). That means a grid of lines (100 nm line distance) was placed over the images via the grid plugin tool from open-source software ImageJ (version 1.52). The orthogonal distance from the clearly visible cell membrane cut by a line to the end of the cThO_2_ stained glycocalyx was measured using the lining tool from ImageJ (Fig. 1e and f). Cut membranes that were not clearly identifiable due to orientation or overlap within the section were not included in the analysis. If there was more than one possible direction due to undulating shape, the shortest path was followed. In total, we measured a minimum of 100 counting events in each human and mouse sample for all treatment groups (control, HEP, PLY).

Data analyses were performed using GraphPad Prism version 9 (GraphPad Software, USA). Distribution of data was assessed with the Shapiro–Wilk test. Normally distributed data were compared with two-way analysis of variance (ANOVA) followed by the Sidak–Holm post hoc test. Data not normally distributed were analyzed by the Kruskal–Wallis test followed by Dunn’s post hoc test. A *P* value < 0.05 was considered statistically significant.

**Fig. 2:**
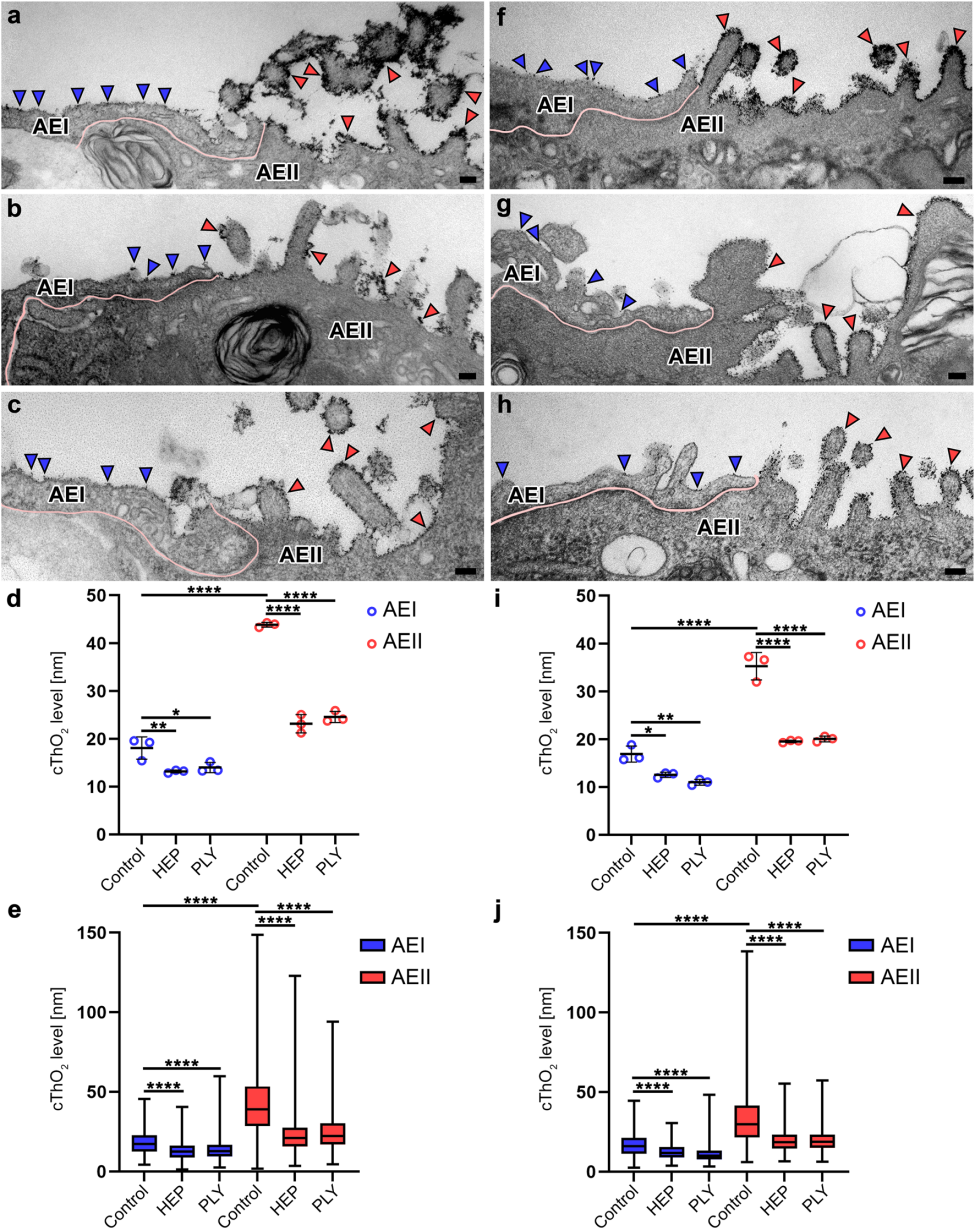
Comparison of cThO_2_ particle levels on control versus HEP- or PLY-treated AEI and AEII. TEM of ultra-thin sections from human lung tissue explants (**a**–**c**) and mouse lungs (**f**–**h**). **a**–**c** and **f**–**h** Boundary between AEI and AEII marked by pink line. CThO_2_ particles on alveolar epithelium marked by blue (AEI) and red (AEII) arrowheads. Compared to control (**a** and **f**), HEP (**b** and **g**) and PLY (**c** and **h**) treatments show a decreased density of cThO_2_ particles on both AEI and AEII with a rather patchy cThO_2_ layer, particularly pronounced in human lung tissue. Scale bars: 100 nm. **d** and** i** Statistical analysis for measured means of human (**d**) and mouse (**i**) treatment subgroups was performed with two-way ANOVA test. Data are shown as mean ± standard deviation. *P < 0.05, **P < 0.01, ****P < 0.0001 compared to controls. **e** and **j** Distribution of all measurements per treatment group shown as box plot with median for human (**e**) and mouse (**j**) samples. Statistical analysis was performed with the Kruskal–Wallis test. ****P < 0.0001 compared to controls

**Fig. 3:**
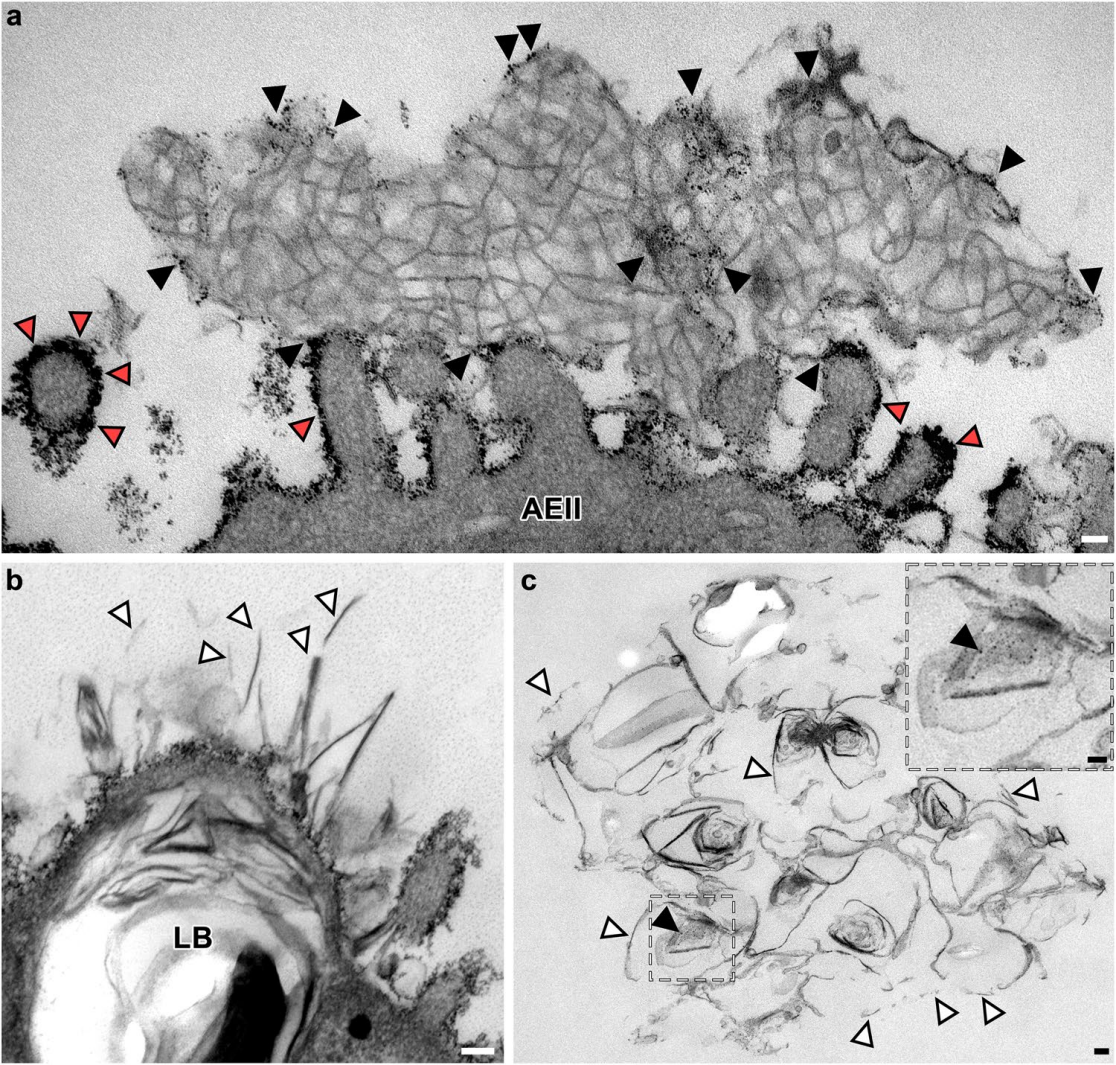
CThO_2_ particles on alveolar surfactant subtypes. TEM of ultra-thin sections from mouse (**a**) and human (**b**–**c**) lung tissue. **a** Control AEII microvilli extending into alveolar lumen containing tubular myelin as intra-alveolar surfactant subtype. Note cThO_2_ particles located not only on the microvilli membranes (red arrowheads) but also in direct relation to tubular myelin (black arrowheads). Scale bar: 50 nm. **b** HEP-treated AEII with exocytosed surfactant components, which appear fragmented (white arrowheads). LB = lamellar body. Scale bar: 100 nm. **c** Fragmented and disorganized surfactant components after PLY treatment (white arrowheads). Individual cThO_2_ particles (black arrowhead) are visible in the magnification on the upper right. Scale bar: 100 nm

The software programs GraphPad Prism version 9 and Adobe Photoshop version 2021 (Adobe Systems Software Ireland Limited, Republic of Ireland) were used to create figures and graphics (Figs. 1, 2, and 3).

### ET and 3D analysis

After 20 min pre-irradiation of the regions of interest (apical human AEII membranes), tilt series of these regions were acquired with a Tecnai G2 TEM (FEI, Eindhoven, Netherlands) with integrated software Tecnai User Interface (version 4.6) with Xplore3D^TM^ software and TEM Imaging and Analysis (version 4.7 SP1) at 200 kV and ×55.000 on-microscope magnification and automatically recorded with a 2 K CCD camera (Olympus SIS, Münster, Germany) (binning 1) in a range from 60° to −60° in steps of 1°. To reduce the common "missing wedge" artifacts (Paavolainen et al. 2014; Koster et al. 1997; Donohoe et al. 2006), we recorded all tilt series from two perpendicular tilt axes (dual-axis ET).

The open-source software package IMOD (version 4.11) was used for further processing of raw ET data. IMOD's etomo was used for 3D reconstruction of the dual-axis tilt series. The calculation was done with an R-weighted back-projection algorithm of the aligned stack. The original tomogram pixel size of 1.84 nm in the *x*-, *y*-, and *z*-direction was maintained for the subsequent analyses. Analyses of cThO_2_ particle density (≙ stained glycosaminoglycan density in three dimensions) were performed using IMOD's 3dmod modeling and display program. Technical details of tomogram generation and tools used for analysis, especially the automatic isosurface, are described in detail on the IMOD homepage: https://bio3d.colorado.edu/imod/ (last accessed March 10, 2023).

**Fig. 4:**
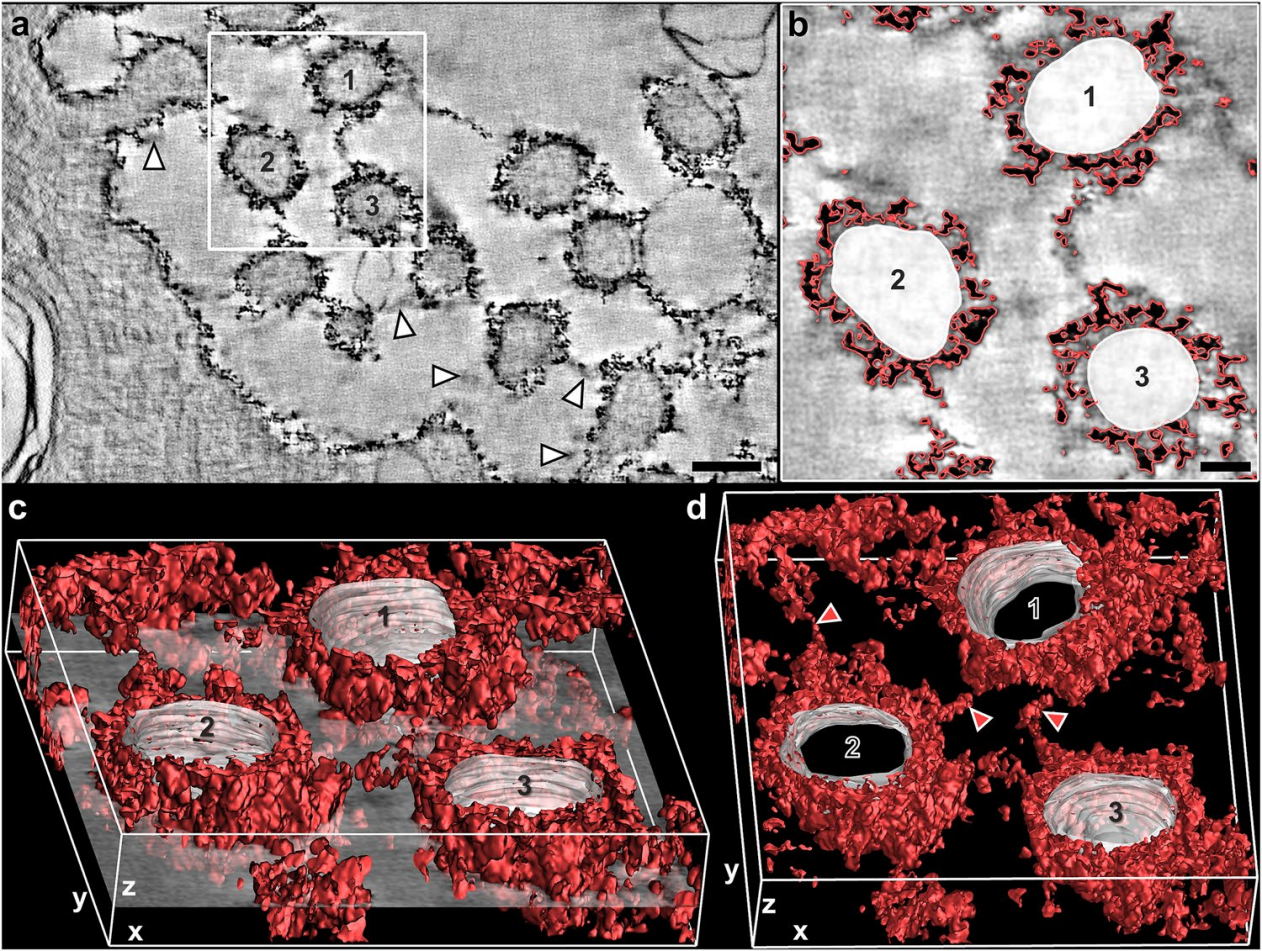
Computer-based cThO_2_ signal analysis of human control AEII. Dual-axis ET of a semi-thin section from human control lung. **a** Single virtual *xy*-slice (*z* = 1.84 nm) out of the tomogram. Note the relatively continuous cThO_2_ particle layer present even in such very thin slice. White arrowheads exemplarily mark “shadow-like” effects in close proximity to the glycocalyx, radiating from the strong signal of cThO_2_ particles from preceding and following virtual slices. Scale bar: 150 nm. **b** Virtual *xy*-slice cutout from **a**. Automatic isosurface for intensity values of cThO_2_ particle outer limits (red) and manually marked area of three microvilli cytoplasm and membranes (1, 2, 3). Scale bar: 50 nm. **c** Virtual transparent *xy*-slice cutout from b rotated around the *y*-axis with resulting 3D model of 55 consecutive above and below virtual *xy*-slices (≈ 100 nm *z*-thickness) in which cThO_2_ particles and microvilli membranes were tracked as labeled in **b**. **d** 3D model of cThO_2_ particles from **c** rotated around the *y*-axis reveals interconnections between microvilli (red arrowheads)

**Fig. 5:**
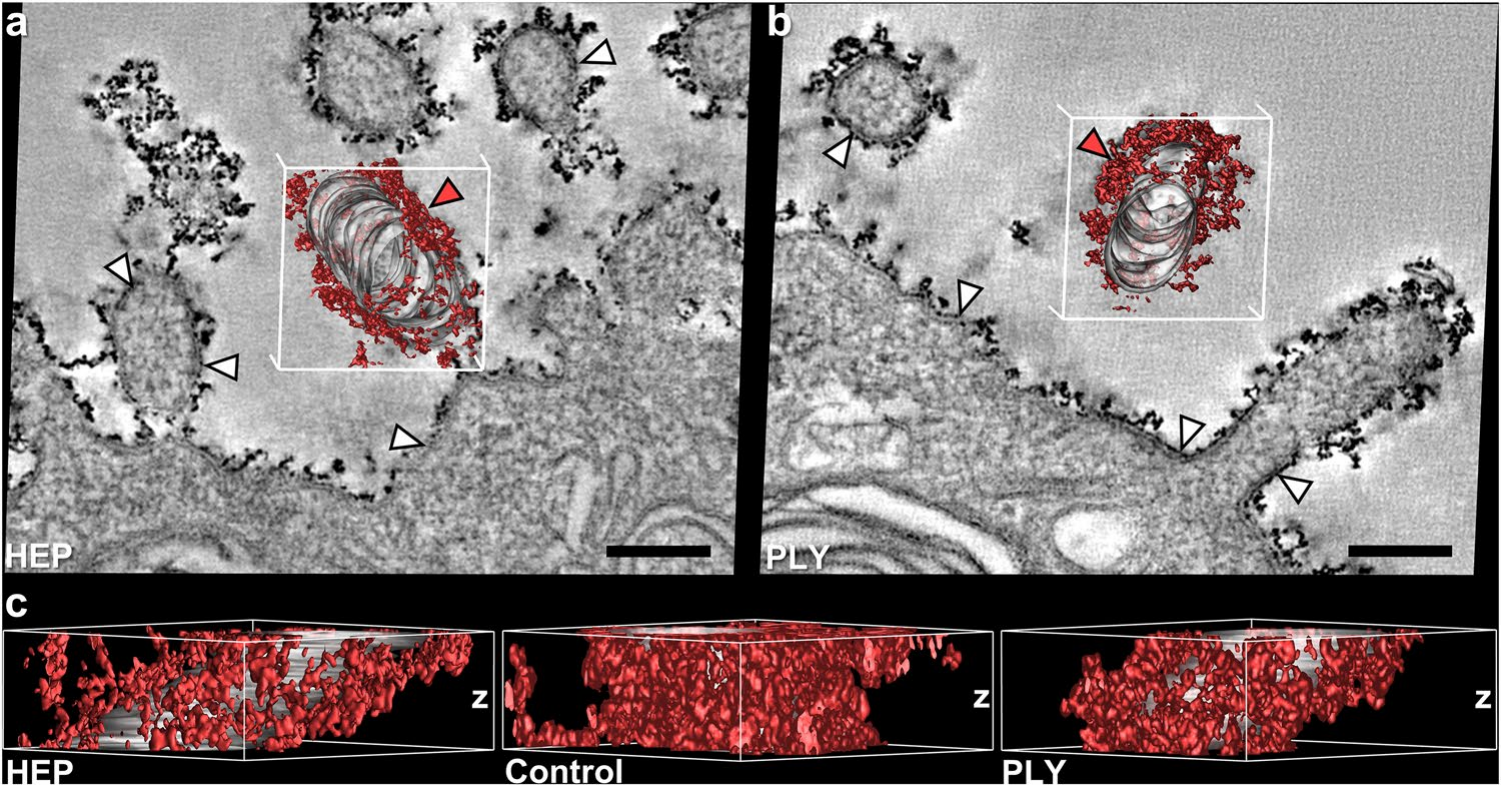
Comparison of cThO_2_ particle distribution in the *z*-direction between control versus HEP and PLY treatment. Dual-axis ET of semi-thin sections (microtome set *z*-thickness = 250 nm) from human HEP- or PLY-treated lung tissue. **a**, **b** Single virtual *xy*-slices (*z* = 1.84 nm) out of the tomogram each with a red 3D cThO_2_ particle model on microvillus membranes (gray) tracked in over 55 consecutive virtual *xy*-slices (≈ 100 nm *z*-thickness) as shown in Fig. 3. Note the more discontinuous cThO_2_ layers compared to Fig. 4a. White arrowheads exemplary mark larger membrane areas on which cThO_2_ staining is absent. Membranes after HEP (**a**) and PLY (**b**) treatment are comparably intact. Red arrowheads on the 3D model mark regions of higher cThO_2_ accumulation whose *z*-distribution is analyzed in c. Scale bars: 150 nm.  **c** View on the *z*-axis of the 3D cThO_2_ particle models from **a** and **b** from the direction of the red arrowheads and for comparison view on the *z*-axis of control microvillus 1 from Fig. 4. HEP and PLY show alterations in the form of cThO_2_ staining gaps of different sizes towards the cell membrane compared to only very small gaps in the control

Microsoft PowerPoint version 2016 (Microsoft Corporation, USA) was used to create figures (Fig. 4 and Fig. 5).

## Results

The following results refer to all electron-dense particles on the alveolar epithelium, which can be assumed to be glycocalyx GAG-bound cThO_2_ particles. CThO_2_ particles were recognizable in all analyzed samples.

### Stereological analysis of alveolar epithelial cThO_2_ particle levels

All apical alveolar epithelial cell membranes in human and mouse control samples showed a thin cThO_2_ particle layer (Fig. 1a, b). On closer inspection, a distinction in the level and density of cThO_2_ particles was already evident in the qualitative examination, as AEI exhibited fewer cThO_2_ particles in the *xy*-dimension compared to AEII (Fig. 1b–d). Measurements of cThO_2_ particle levels as orthogonal intercepts for quantitative analysis (see methods section) is exemplified in Fig. 1e, f. All measured mean values (with standard deviation) listed below are summarized in Table 1. The average level of cThO_2_ particles was for control human AEI 18.10 nm (2.33 nm) and mouse AEI 16.92 nm (1.69 nm), for human AEII 43.85 nm (0.50 nm) and for mouse AEII 35.33 nm (2.88 nm) (Fig. 2a, d, f, and i). While the cThO_2_ particle level on human and mouse AEI was relatively continuous, there were locally larger extensions on AEII cells, especially microvilli-associated with measurements of up to a 149 nm cThO_2_ particle level in human and 138 nm in mouse control samples (Fig. 2e and j).

**Table 1:** Measured mean values with standard deviation (SD)

	Control AEI	HEP AEI	PLY AEI	Control AEII	HEP AEII	PLY AEII
*Human*						
Mean cThO_2_ level sample 1 (SD) [nm]	19.60 (8.48)	12.85 (6.15)	15.23 (8.76)	43.30 (22.03)	23.12 (13.50)	25.95 (10.33)
Mean cThO_2_ level sample 2 (SD) [nm]	19.29 (7.16)	13.27 (4.99)	13.33 (6.13)	43.99 (21.10)	21.28 (7.99)	24.14 (12.37)
Mean cThO_2_ level sample 3 (SD) [nm]	15.42 (6.99)	13.42 (6.18)	13.45 (5.32)	44.26 (20.47)	25.11 (13.34)	23.75 (12.87)
Average of means (SD) [nm]	18.10 (2.33)	13.18 (0.29)	14.00 (1.06)	43.85 (0.50)	23.17 (1.92)	24.62 (1.18)
*Mouse*						
Mean cThO_2_ level mouse 1 (SD) [nm]	16.14 (6.82)	12.82 (4.47)	10.39 (4.42)	32.02 (20.47)	19.67 (7.97)	19.46 (7.18)
Mean cThO_2_ level mouse 2 (SD) [nm]	15.76 (6.83)	11.95 (5.03)	11.05 (4.20)	37.31 (10.14)	19.30 (6.69)	20.64 (6.98)
Mean cThO_2_ level mouse 3 (SD) [nm]	18.85 (8.16)	12.92 (4.69)	11.59 (5.85)	36.64 (22.87)	19.76 (7.17)	20.16 (8.06)
Average of means (SD) [nm]	16.92 (1.69)	12.56 (0.54)	11.01 (0.60)	35.33 (2.88)	19.58 (0.24)	20.09 (0.59)

Both treatments resulted in significant reduction of cThO_2_ particle levels (Fig. 2b–e and g–j). There was a reduction of about 47% (HEP) and 44% (PLY) on human AEII and about 45% (HEP) and 43% (PLY) on mouse AEII. The effect was somewhat less pronounced on AEI with a reduction of about 27% (HEP) and 23% (PLY) on human AEI and about 26% (HEP) and 35% (PLY) on mouse AEI. Values after treatment: human AEI 13.18 nm (0.29 nm) (HEP) and 14.00 nm (1.06 nm) (PLY); mouse AEI 12.56 nm (0.54 nm) (HEP) and 11.01 nm (0.60 nm) (PLY); human AEII 23.17 nm (1.92 nm) (HEP) and 24.62 nm (1.18 nm) (PLY); mouse AEII 19.58 (0.24 nm) (HEP) and 20.09 nm (0.59 nm) (PLY). In addition, as qualitative observation, the HEP and PLY groups showed a decrease in the density of cThO_2_ particles on both AEI and AEII. At lower magnifications on the microscope, the cThO_2_ layer appeared rather punctate or patchy, with individual cThO_2_ particles visible only at higher magnifications for the measurements. This effect was particularly pronounced in the human samples.

We also detected cThO_2_ particles on intra-alveolar surfactant subtypes, including tubular myelin, in both human and mouse samples (Fig. 3a). In addition, the HEP- and PLY-treated samples showed partially fragmented and disorganized surfactant components (Fig. 3b and c).

### 3D analysis of cThO_2_ particle distribution via dual-axis ET

To better understand the quantitative *xy*-dimension measurements in the *z*-dimension and the qualitative finding of different cThO_2_ particle densities under different alveolar conditions, we exemplarily analyzed the 3D distribution of cThO_2_ particles on apical AEII membranes in human control, HEP and PLY semi-thin sections. Dual-axis data generation and analysis turned out to be distinctly better than single-axis analysis. In the latter, cThO_2_ particle aggregates appeared comparatively blurred and skewed. In contrast to the conventionally analyzed ≈ 70 nm thick sections, the ≈ 2 nm thick virtual tomogram slices exhibited “shadow-like” effects at many locations in close proximity to the glycocalyx (Fig. 4a). This phenomenon was caused by the strong signal of cThO_2_ particles radiating into the preceding and following virtual slices. Due to the different electron density from the very electron-dense cThO_2_ particles that was no obstacle for 3D model generation.

IMOD’s isosurface function was used to create computer-based cThO_2_ particle signal analysis (Fig. 4b, c). The models of human control AEII showed an extensive cThO_2_ particle distribution with many interconnections which can be observed between microvilli (Fig. 4d) and only few very small gaps to the cell membrane. In contrast, virtual tomogram slices and 3D models after HEP (Fig. 5a) and PLY (Fig. 5b) treatment looked somewhat frayed as in the ultra-thin sections. A reduced cThO_2_ particle density after the treatment, which was qualitatively observed in the conventional evaluation in the *xy*-dimension, was confirmed in the *z*-dimension (Fig. 5c). A reduced cThO_2_ particle density became more apparent. Compared to the control model, HEP and PLY models showed changes in the form of many cThO_2_ gaps of different sizes towards the cell membrane. Considering the altered cThO_2_ model under PLY, we examined areas with well-preserved membranes in virtual sections more closely for possible pores and other membrane defects that would explain GAG loss. However, membranes with absent cThO_2_ particles appeared intact as well (Fig. 5b).

## Discussion

Our study provides quantitative and qualitative electron microscopic data on the cell-specific alveolar epithelial glycocalyx GAG distribution and shows that specific HEP-induced GAG shedding is associated with a decrease in cThO_2_ particle level and density. We found a comparable GAG shedding under the influence of PLY, for which the exact mechanism remains to be elucidated.

The present investigation is a comparative evaluation based on cThO_2_ particle staining patterns. Like cThO_2_, many conventional glycocalyx stainings rely on electrostatic interactions that mark GAG components only, the glycocalyx, however, is neither complete nor specific like immunocytochemistry. The native glycocalyx expansion may differ as well as staining patterns of other methods, which should be further studied. In addition, superposition artifacts must be considered for measurements in both native and fixed stained sections when using conventional TEM analyses. Due to the section thickness and orientation, several glycocalyx layers are measured in the *z*-direction. Moreover, not all these glycocalyx layers are oriented perpendicular to the visualized membrane surface given the isotropic alignment of lung tissue. These non-perpendicular perspectives cause the cThO_2_ particle level to appear longer than it actually is. Correction factors for determining the actual length from orthogonal intercepts of microscopic sections have already been determined for measuring membrane thickness (Jensen et al. 1979). This does not change statistics, but such factors become important and have to be developed for studies on exact expansion of native glycocalyx. However, it must be considered that to date, no microscopical method can provide results that are not distorted in some way by the preparation (i.e., embedding, cutting, beam).

ET partly avoids the above described superposition artifacts by achieving a resolution of ≈ 2 nm in all three dimensions. Using this technique, it is important to consider that the long electron beam illumination of the tilt series causes tissue shrinkage in the *z*-direction, leading to a compressed appearance of the particles in the *xz*- and *yz*-direction (Luther 2006) but not in the *xy*-direction, if the section is well attached to the film (Luther 2006; Laue et al. 2021). However, since the number of bound cThO_2_ particles does not change, qualitative analysis of densities is perfectly feasible. The size and electron density of cThO_2_ proved to be excellent properties for computer-based generation of 3D distribution models. Analyzing these models, we determined that glycocalyx shedding is structurally reflected not only in the quantified reduction of cThO_2_ particle levels but also in a reduction of cThO_2_ particle density. Including density values, measured HEP and PLY treatment damage may reach higher percentage values and should be considered in future studies. In addition, the methodological impact on shedding rates should be further investigated. Thus, mice treatment with HEP resulted in significant GAG detachments even at much lower concentrations than used in other studies (Rizzo et al. 2022; Haeger et al. 2018). In the literature, data on the range of activity and corresponding structural and functional alveolar glycocalyx effects are missing so far.

CThO_2_-stained GAG levels in human and mouse lungs measured in this study are significantly lower in AEI compared with AEII. Earlier investigations, including a study by our group, already indicated a different staining pattern between these two cell types in qualitative analyses (Kuhn 1968; Adamson and Bowden 1970; Roth 1973; Ochs et al. 2020). The functional reason for lower level of glycocalyx GAGs on AEI has not been investigated yet, but a major factor might be gas exchange, which must be provided by flat AEI covering ≈ 95% of the alveolar surface. The opposite endothelial glycocalyx in pulmonary capillaries is also very thin relative to other capillaries, supporting the theory of diffusion function (Suzuki et al. 2022). In contrast, cuboidal AEII located in the alveolar corners have secretion and renewal functions (Ochs et al. 2020). Studies on the individual distribution of glycocalyx subcomponents on AEI and AEII in health and disease, including fetal and senescent time points, could therefore be of potential value to elicit possible cell type-specific functions. It is further to mention that the pathophysiological condition of chronic obstructive pulmonary disease was reported to be accompanied by elevated endogenous heparanase expression (Morris et al. 2015). Considering the affected human lungs used in our study, AEI and AEII GAG expansion could differ from completely healthy lungs. The slightly higher control AEI and AEII measurements of human compared to mouse lung tissue might be related, in part, to the fact that human lung alveoli are also larger than those of mice (Irvin and Bates 2003; Vignero et al. 2018). The gender could impact measurements as well. The present data are exclusively based on female human and mouse lungs. Rizzo et al. (2022) recently found increased GAG shedding in patients with acute respiratory distress syndrome in the airspace to be associated with male gender.

In contrast to the epithelial glycocalyx, the endothelial glycocalyx has been previously recognized and studied as an essential structure for endothelial function, with alterations described in various acute and chronic diseases (Foote et al. 2022; Patterson et al. 2022; Uchimido et al. 2019; Masola et al. 2021; Puchwein-Schwepcke et al. 2021). However, there is growing evidence that the alveolar epithelial glycocalyx plays an important role in regulating inflammation, infection, and allergic processes (Chignalia et al. 2016) and that acute lung injury is associated with alveolar glycocalyx degradation (Rizzo et al. 2022; Haeger et al. 2016, 2018; LaRiviere et al. 2020). We found alveolar epithelial glycocalyx GAG shedding under subcytolytic PLY dosages with similar patterns to HEP-induced shedding. Whether PLY predominantly impacts on heparan sulfate detachment as well, or other GAGs and anchoring proteins such as sydecan-1 might be addressed, needs to be explored by specific stainings. Likewise, effects of different PLY concentrations upon the underlying processes are to be examined. Alveolar glycocalyx degradation during intratracheal lipopolysaccharide-induced lung injury has been previously described, whereby the release of heparan sulfate from the epithelium was discussed to inhibit the spread of infection (Haeger et al. 2018). A mechanism of lipopolysaccharides was shown to be the induction of matrix metalloproteinase expression, capable of cleaving proteoglycans (Zhang et al. 2021; Rizzo and Schmidt 2023). *Streptococcus pneumoniae*, like other bacteria, uses heparan sulfate as a receptor to adhere to host cells and infect them (Rajas et al. 2017; Bernfield et al. 1999; Aquino et al. 2022). GAG antagonists have been shown to inhibit such microbial attachment and invasion of host cells in vitro and decrease virulence in vivo (Bartlett and Park 2010). In addition, soluble heparan sulfate may function as a decoy receptor (Haeger et al. 2018; Chandra et al. 2019). In this context, recent research investigates the utility of heparin to remove pathogens from the blood, as many bacteria bind to heparin similarly to heparan sulfate (Seffer et al. 2021). Furthermore, endothelial glycocalyx degradation via heparanase activation has already been demonstrated in the pathophysiological process of acute lung injury (Schmidt et al. 2012; Li et al. 2020), in which an aberrant epithelial heparanase expression might be assumable as well. Moreover, it has recently been shown in vitro that PLY can induce the release of the GAG hyaluronan from human lung endothelial cells in response to the production of reactive oxygen species (Sauer et al. 2022). Finally, interactions of PLY with the phospholipid bilayer (Nollmann et al. 2004) could disrupt the integrity of GAGs within the membrane, leading to shedding.

As previously described (Ochs et al. 2020), we observed cThO_2_ particles on surfactant subtypes in the alveolar hypophase. Specific staining of glycocalyx subcomponents should follow to reliably identify potential glycocalyx–surfactant interactions. A feasible GAG candidate would be hyaluronan whose polar heads are thought to attract surfactant phospholipids and interact with the hydrophobic surfactant proteins B and C (Bray 2001). Studies indicate improved biophysical activity of surfactant by this GAG (Souza-Fernandes et al. 2006; Lu et al. 2005a, 2005b; Lopez-Rodriguez et al. 2013). Heparan sulfate is conceivable as well, since heparin as a highly sulfated form of heparan sulfate can bind to surfactant proteins A, B, and D, and selective GAG shedding by HEP has been described sufficient to induce surfactant dysfunction in mice (Rizzo et al. 2022). Further candidates of interest are the hydrophilic surfactant proteins A and D which may interact with the glycocalyx via a specific carbohydrate recognition domain possibly influencing their primarily immunomodulatory functional capacities (Ochs et al. 2020).

In conclusion, our study demonstrates a glycocalyx GAG layer on the alveolar epithelium that is morphologically distinguishable between AEI and AEII. In humans and mice, the glycocalyx GAGs of both cell types respond to lung injury induced by HEP and PLY in a similar pattern with a loss of GAG height and density. There is thus a need to better understand the underlying cell type-specific distribution of glycocalyx subcomponents to achieve a comprehensive understanding of physiological and pathophysiological conditions.

## Data Availability

The datasets generated and analyzed during the current study are available from the corresponding authors on reasonable request.
